# Natural Bioactive Products with Antioxidant Properties Useful in Neurodegenerative Diseases

**DOI:** 10.1155/2019/7151780

**Published:** 2019-05-09

**Authors:** Francisco J. B. Mendonça-Junior, Marcus T. Scotti, Anuraj Nayarisseri, Ernestine N. T. Zondegoumba, Luciana Scotti

**Affiliations:** ^1^Postgraduate Program in Natural and Synthetic Bioactive Products, Federal University of Paraíba, João Pessoa, PB, Brazil; ^2^Laboratory of Synthesis and Drug Delivery, Department of Biological Science, State University of Paraiba, João Pessoa, PB, Brazil; ^3^In Silico Research Laboratory, Eminent Biosciences, Indore, 452010 Madhya Pradesh, India; ^4^Bioinformatics Research Laboratory, LeGene Biosciences Pvt. Ltd., Indore, 452010 Madhya Pradesh, India; ^5^Department of Organic Chemistry, Faculty of Science, University of Yaoundé I, PO Box 812, Yaoundé, Cameroon; ^6^Teaching and Research Management-University Hospital, Federal University of Paraíba, João Pessoa, PB, Brazil

Neurodegenerative diseases (NDs) constitute a large group of pathological conditions, characterized by a progressive loss of neuronal cells, which compromise motor and/or cognitive functions. The most common NDs are Alzheimer's disease (AD), amyotrophic lateral sclerosis (ALS), Parkinson's disease (PD), and Huntington's disease (HD). The causes of these pathologies are multifactorial and not fully understood, but it is well known that factors related to aging and to the overproduction of free radical and reactive oxygen species lead to oxidative stress and to cell death, which are extremely related. Whereas oxidative stress plays an unquestionable and central role in NDs, the control of free radicals and reactive oxygen species levels represents an interesting and promising strategy to delay neurodegeneration and attenuate the associated symptoms.

In this context, several natural bioactive compounds isolated from plants, fungi, and algae, among others, and also synthetic compounds inspired by natural scaffolds, which present antioxidant properties, including vitamins C and E, anthocyanins, and phenolic compounds, are extensively described as potential palliative agents of neurodegenerative symptoms. *In vitro* and *in vivo* studies, performed with extracts and fractions of plants and with isolated natural bioactive compounds, provide evidence of the role of these substances in the modulation of the cellular redox balance and in the reduction of the formation of reactive oxygen species originating from oxidative stress, thereby demonstrating their great value as antioxidant agents and cellular protectors.

In this special issue, articles were selected that address new therapeutic alternatives on the antioxidant and anti-inflammatory role and the consequent neuroprotetor of natural (or inspired) bioactive compounds in the prevention/treatment or improvement of neurodegenerative diseases. This special issue compiles fifteen (15) manuscripts including three (3) reviews and twelve (12) research papers, which show recent research about the discovery of plant-derived antioxidants with application in neurodegenerative diseases.

The review by R. Avila-Sosa et al. describes the antioxidant effects of main bioactive components isolated from Amazonian fruits. Among other activities, the authors highlight antioxidants, immunomodulatory, anticancer, anti-inflammatory, and antidepressant properties of phenolic compounds, unsaturated fatty acids, carotenoids, phytosterols, and tocopherols.

The review by X. Zhao et al. highlights the benefits of vitamin supplementation in the treatment or improvement of the clinical symptoms of Parkinson's disease. The authors summarized the biological correlations between vitamins and PD as well as the underlying pathophysiological mechanisms, demonstrating that the antioxidant properties and the regulatory gene expression promoted by vitamins are beneficial for the treatment/prevention of PD.

Due to the fact that many diseases that affect the central nervous system also promote blood-brain barrier (BBB) destruction, consequently increasing BBB permeability, in the third review, Z. Chen et al. carry out a systematic review of about the evidence of possible neuroprotective borneol (terpenoid) effects for ischemic stroke. The authors have found much evidence that borneol exerted a significant decrease of BBB permeability, thus acting as a neuroprotector.

Ten of the eleven research articles deal with the proof of antioxidant, anti-inflammatory, and neuroprotective activities in *in vitro* and/or *in vivo* models, of plant and/or cyanobacteria extracts, and natural products isolated or chemically modified. The only article that eludes this theme is the work of A. F. M. Monteiro et al. which carried out *in silico* studies aimed at the identification of potentially useful flavonoids for *in vitro*/*in vivo* screening in Parkinson and Alzheimer models.

G. Oboh et al.'s group found that the alkaloid extract from the African Jointfir (*Gnetum africanum*) is capable to counteract the Mn-induced elevation in AChE activity, NO, and ROS levels. I. K. Martins et al. observed the neuroprotective effect of the methanolic fraction of *Anacardium microcarpum* (from Brazil). This fraction was able to prevent neurodegeneration through the chelating properties toward ROS species, which is dependent on ERK1/2 and AKT phosphorylation; however, it does not prevent mitochondrial damage by 6-OHDA.

K. Adamczyk et al. evaluated the antihyaluronidase, antiacetylcholinestarase, and anti-DPPH activities of several *Eleutherococcus* species cultivated in Poland. The methanolic extract was shown to be rich in polyphenols and promoted a reduction in DPPH in a time-dependent mode. *E. gracilistylus* and *E. sessiliflorus* showed the highest inhibition of AChE, and *E. henryi* was the best hyaluronidase inhibitor. R. B. de Oliveira Caland et al. observed the neuroprotective and antioxidative effect of pasteurized orange juice (*Citrus sinensis L*.) rich in carotenoids. The authors observed reduction in ROS production and upregulation of the expression of antioxidant and chaperonin genes, generating greater resistance to oxidative stress.

I.-C. Chen et al. evaluated the neuroprotective effects of formulated Chinese herbal medicines in a cell model of tauopathy. Shaoyao Gancao Tang (*P. lactiflora* and *G. uralensis* in a 1 : 1 ratio) presented the best antioxidative and anti-inflammatory results, reducing the tau misfolding and the production of the reactive oxygen species (ROS) level, especially nitric oxide (NO). In the research article by D. Nuzzo et al.'s group, the authors observed the neuroprotective effect of the cyanobacteria extract (Klamin®). Klamin® interferes with A*β* aggregation kinetics, exerts a protective role against beta amyloid (A*β*), and promotes activation of IL-6 and IL-1*β* inflammatory cytokines.

Y.-J. Wang et al. observed the antioxidant and neuroprotective activities of the extract of *Centipeda minima* and four isolated sesquiterpenoids. They found that the extract reduces glutamate and *tert*-butyl hydroperoxide-induced neuronal death, ROS production, and mitochondria dysfunction. Among the isolated sesquiterpenoids, 6-*O*-Angeloylplenolin and arnicolide D were the most active and responsible for the activation of the Nrf2 pathway and inhibition of ROS production. The study conducted by K. K. S. Narasimhan et al. has demonstrated that scopoletin (one of the main components from *Morinda citrifolia*) prevents oxidative injury and mitigates protein aggregation by the markedly upregulated DJ-1/Nrf2/ARE pathway.

L. Subedi et al. observed the antioxidant and anti-inflammatory effects of sulforaphane-enriched broccoli sprouts (SEBS) which lead to their neuroprotective effects. SEBS has protective effects of neuroinflammatory conditions by inhibition of the LPS-induced activation of the NF-*κ*B signaling pathway, by the secretion of inflammatory proteins (inhibition of inflammatory cascade), and least by the upregulation of the expression of Nrf2 and HO-1, improving the scopolamine-induced memory impairment in mice. Y. Lee et al. verified that *γ*-mangostin (one of the major constituents from *Garcinia mangostana* fruits) reduces the oxidative neurotoxicity through the inhibition of H_2_O_2_-induced DNA fragmentation, ROS generation, lipid peroxidation, and DPPH radical formation, which is associated with the protection against H_2_O_2_-induced oxidative neuronal death. Orally, *in vivo*, *γ*-mangostin also improved scopolamine-induced memory impairment in mice. And finally, J.-S. Ye et al. observe the neuroprotective effect of Honokiol (a lignan isolated from the *Magnolia* genus) in postoperative cognitive change. Honokiol-mediated mitophagy inhibits the activation of the NLRP3 inflammasome and neuroinflammation in the hippocampus by increasing the expression of LC3-II, Beclin-1, Parkin, and PINK-1 at protein levels and through attenuation of mitochondrial structure damage and reduction of mtROS and MDA generation.

This compilation of articles gives us an up-to-date sample of the therapeutic potential of natural products in providing potential drugs and/or plant candidates to treat, prevent, or ameliorate the oxidative stress associated with neurodegenerative diseases including, but not limited to, Parkinson's and Alzheimer's diseases. We are sure that the information available in this issue will be very useful and will contribute to the future success of new therapies for neurodegenerative diseases.

